# Antiparasitic activities of hydroethanolic extracts of *Ipomoea imperati* (Vahl) Griseb. (Convolvulaceae)

**DOI:** 10.1371/journal.pone.0211372

**Published:** 2019-01-25

**Authors:** Ana Cássia M. Araujo, Eduardo B. Almeida Jr., Cláudia Q. Rocha, Aldilene S. Lima, Carolina R. Silva, Marcelo M. P. Tangerina, José S. Lima Neto, Lívio M. Costa-Junior

**Affiliations:** 1 Programa de Pós-Graduação em Biodiversidade e Conservação, Centro de Ciências Biológicas e da Saúde, Universidade Federal do Maranhão, São Luís, Maranhão, Brazil; 2 Departamento de Biologia, Universidade Federal do Maranhão, São Luís, Maranhão, Brazil; 3 Departamento de Química, Universidade Federal do Maranhão, São Luís, Maranhão, Brazil; 4 Departamento de Patologia, Universidade Federal do Maranhão, São Luís, Maranhão, Brazil; 5 Laboratório de Bioprospecção de Produtos Naturais, Instituto de Biociências, Universidade Estadual Paulista, São Vicente, São Paulo, Brazil; 6 Universidade Federal do Piauí, Departamento de Farmácia, Laboratório de Geoquímica Orgânica, Teresina, Piauí, Brazil; Beijing Forestry University, CHINA

## Abstract

*Ipomoea imperati* is widely used in tropical areas to treat several pathological conditions. The effect of this plant against parasitic species has not been investigated even being used for this purpose in the Brazilian northeastern. This study aimed to evaluate the anthelmintic and acaricide potential of a hydroethanolic extract of *I*. *imperati* leaves and stolons. *I*. *imperati* leaves and stolons were crushed and subjected to maceration in ethanol 70% (v/v), after which the solvent was removed using a rotary evaporator. The chromatographic profile of the extract was obtained by UV Spectrum high-performance liquid chromatography and compounds were identified by liquid chromatography/electrospray ionization tandem mass spectrometry. Identification of the compounds present in the extract was achieved by comparing their retention times and UV spectra with data in the literature. Anthelmintic activity was evaluated by larval exsheathment inhibition assays using *Haemonchus contortus* larvae and five concentrations of each extract ranging from 0.07 to 1.2 mg/mL. Acaricide activity was evaluated via larval immersion of *Rhipicephalus microplus* in eight concentrations of each extract ranging from 5.0 to 25.0 mg/mL. Live and dead larvae were counted after 24 hours. The median inhibitory concentration (IC_50_) for *H*. *contortus* larvae and the median lethal concentration (LC_50_) for *R*. *microplus* larvae were calculated. Twelve compounds were observed in the hydroethanolic extract of leaves, with a predominance of the aglycone form of flavonoids and tannins. This extract was effective against *H*. *contortus* larvae, presenting an average inhibitory concentration of 0.22 mg/mL, but showed no activity toward *R*. *microplus* larvae. The stolon hydroethanolic extract presented 11 compounds, with phenolic acids and glycosylated flavonoids prevailing. This extract showed low activity on *R*. *microplus* and no effect on inhibiting *H*. *contortus* larval exsheathment at the concentrations tested. This study is the first to assess the anthelmintic and acaricidal activities of *I*. *imperati*. Data reported confirm promising potential of *I*. *imperati* leaves hydroethanolic extract against *H*. *contortus*. This effect could be due to its secondary compounds presents in this extract, such as procyanidin, kaempferol, isoquercitrin and rutin.

## Introduction

Parasitic infestations impose major limitations to the development of livestock, resulting in substantial losses in global productivity [1]. The tick *Rhipicephalus microplus* (Canestrini, 1887) and the gastrointestinal nematode *Haemonchus contortus* (Rudolphi, 1803) Cobb, 1898 are among the main parasites infecting cattle and small ruminant, respectively [2, 3]. These parasites are prominent in tropical and subtropical areas [4, 5] and cause damage to animals that leads to significant economic loss due to the reduction in productivity as well as to the expense of parasitic control [2, 3, 6].

The use of plants derived from traditional knowledge as a palliative resource of treatment for such infestations is very common in developing countries [7, 8]. Although many plants have already been scientifically validated, there are still many species that have not yet been investigated, mainly in tropical areas where there is a great diversity of available flora [9,10].

*Ipomoea* of the family Convolvulaceae is notable due to its abundant species richness, comprising between 600 and 700 species that are largely concentrated in the Americas [11]. Many of these species are used empirically in the treatment of several pathological conditions, but relevant properties have only been investigated for a few of these species [12]. An important species used in traditional medicine is *Ipomoea imperati* (Vahl) Griseb., popularly known as "salsa", “campainha branca” or "salsa da praia" in Brazil (or “beach morning glory” in other countries), names also used for other species of *Ipomoea* [e.g. *I*. *pes-caprae* (L.) R.Br., *I*. *asarifolia* (Desr.) Roem. & Schult] [13]. A perennial herb that is stoloniferous and halophytic, with a white gamopetalous corolla and oblong leaves, *I*. *imperati* occurs most frequently in the area near the sea, occupying coastal dunes [14, 15, 16, 17].

Rural communities in the Maranhão State, Brazil, have used this plant for antiparasitic purposes, in tick control and diarrhea treatment in ruminants (Costa Jr, personal communication). Based on pharmacological studies, the important therapeutic potential of this species involves antinociceptive [18], anti-oxidative, anti-ulcerogenic [19], anti-spasmodic and anti-inflammatory properties [20, 21]. In the face of the reports of indications of popular use for parasitic control and the lack of experimental verification regarding at antiparasitic property of this species, the present study aims to investigate differences in the chemical composition and effectiveness of extracts of different parts of *I*. *imperati* against the tick *R*. *microplus* and the nematode *H*. *contortus*.

## Materials and methods

The animal experimental procedures were performed in accordance with the guidelines of the Animal Ethics Committee of Federal University of Maranhão and were approved by this committee under protocol number 23115018061/2011-11. The plant material collect were approved by SISBIO (Ministry of the environment, Brazil) under number 45593. The collect did not involve endangered or protected species.

### Plant material

Leaves and stolons of *I*. *imperati* were collected in an area of Restinga (02°28' 23"S, 44°03'13"W), located in the municipality of São José de Ribamar, State of Maranhão, Brazil, in December 2016 (dry season). The plant was herborized and identified, and a sample (voucher specimen—MAR 9180) is deposited at the Herbarium of Maranhão (MAR), located in Federal University of Maranhão, Brazil [22].

### Preparation of extracts

The plant material (leaves and stolons) was dried in air circulation oven (Tecnal, Piracicaba, SP, Brazil) at 40°C and crushed in a knife mill (Tecnal). The resulting material of each plant part was separately subjected to maceration for seven days in 70% (v/v) ethanol. The solution was filtered every 48 hours, and the residue was re-extracted using the same amount of solvent. This procedure was repeated to obtain three extractions. The solvent was removed by evaporation using a rotary evaporator to obtain the hydroethanolic extract [23].

### Chemical characterization

The samples were filtered through a 0.22-μm PTFE (poly-tetrafluoroethene) filter Simplepure and dried at room temperature. The dry extract was diluted to 10 mg/mL in high-performance liquid chromatography (HPLC) solvent. Aliquots of 20 μL were injected directly into an UV Spectrum HPLC (UV-HPLC) with detection at 254 nm. A Shimadzu model HPLC system (Shimadzu, Kyoto, Japan) was used, consisting of a solvent delivery module with a double-plunger reciprocating pump and UV-VIS detector (SPA-10A); a Luna 5-μm C18 100 A (150 μm x 4.6 μm) column was used. The A and B elution solvents were 2% acetic acid in water and methanol, respectively, according to the following gradient: 5% at 100% of B in 60 min. The flow rate was 1 mL/min, and the column temperature was 20°C. Data were collected and processed using LC Solution software (Shimadzu).

The extracts were analyzed using a Shimadzu Prominence liquid chromatography system with two Shimadzu LC-20AD automatic injector (SIL-20A HT) pumps. A C18 Shim-pack XR-ODS (2 mm x 30 mm, 2.2 μm) column was used in the analyses. The mobile phase was acidified ultrapure water (0.05% HCOOH) and HPLC grade methanol, also acidified (0.05% HCOOH), at a flow rate of 0.25 mL/min, with the methanol gradient increasing as follows: 2% methanol in 0–2 min; 10% in 5 min; 20% in 7 min and 100% in 30 min. The injection volume was 5.0 μL. The LC was coupled to a mass spectrometer (Amazon X, Bruker, Massachusetts, USA) equipped with electrospray ionization (ESI) and an ion-trap (IT) type analyzer in negative mode, under the following conditions: 5 kV capillary voltage, capillary temperature 220°C, entrainment gas (N2) flow 8 L/min, nitrogen nebulizer pressure at 10 psi. The acquisition range was *m/z* 100–1000, with two or more events. Standards at 50 ppm were included.

Direct flow infusion of the samples was performed with a Thermo Scientific LTQ XL linear ion trap analyzer equipped with an electrospray ionization (ESI) source, in negative mode (Thermo, San Jose, CA, USA). A stainless-steel capillary tube at 280°C, spray voltage of 5.00 kV, capillary voltage of -90 V, tube lens of -100 V and 5 μL/min flow were applied. Full scan analysis was recorded in *m/z* ranging from 100–1000. Multiple-stage fragmentations (ESI-MS^n^) were performed using the collision-induced dissociation (CID) method against helium for ion activation. The first event was a full-scan mass spectrum to acquire data on ions in that *m/z* range. The second scan event was an MS/MS experiment performed by using a data-dependent scan of [M-H]^-^ molecules from the compounds of interest at a collision energy of 30% and an activation time of 30 ms. Product ions were then subjected to further fragmentation under the same conditions until no more fragments were observed. Identification of the different compounds in the chromatographic profiles of the hydroethanolic extracts was performed by comparing their retention times and UV spectra with literature data.

### Biological assays

#### Anthelmintic test

Third-stage larvae (L_3_) were obtained from a donor sheep with a monospecific experimental infection of *H*. *contortus* isolated from a naturally infected goat. The assay for larval exsheathment inhibition was performed as previously described by Bahuaud et al. [24]. Five concentrations were prepared for each of the two extracts (leaves and stolons): 1.2, 0.6, 0.3, 0.15 and 0.07 mg/mL, all diluted in 2% methanol. The negative control was 2% methanol. Briefly, the L_3_ larvae were incubated in the different concentrations for 3 h at 22°C, after which the larvae were washed with phosphate-saline buffer (PBS) and centrifuged (2,540 x *g*) three times. Approximately 1,000 larvae/tube were subjected to artificial exsheathment by contact with sodium hypochlorite (2.0%, w/v) and sodium chloride (16.5%, w/v). Four replicates were performed per concentration. The kinetics of larval exsheathment in the different experimental treatments was then monitored at 0, 20-, 40- and 60-min intervals by microscopic observations (40×).

#### Acaricidal test

Engorged *R*. *microplus* females susceptible to all known synthetic chemical acaricides (8^th^ generation of Porto Alegre strain maintained in the laboratory by experimental infestation) were collected from calves experimentally infested with and without recent contact with chemical acaricides. The engorged females collected were washed in water and maintained in the laboratory at 27°C and ≥80% relative humidity (RH) until oviposition was completed. The 14 to 21-day-old larvae resulting from those eggs were used for the larval immersion test.

The larval immersion test was performed according to the method of Klafke et al. [25]. The extracts (leaves and stolons) were diluted in 70% ethanol at eight concentrations ranging from 5.0 to 25.0 mg/mL. A 1-mL aliquot of each concentration was transferred to 1.5-mL tubes, and approximately 500 tick larvae were placed in each tube; 70% alcohol was used as the control. Immediately after addition of the larvae, the tube was closed, and the mixture was vigorously agitated. After 10 min, the larvae were transferred to a filter paper to dry. After drying, approximately 100 larvae were transferred to a clean dry filter paper (8.5 × 7.5 cm) that was folded and closed with clips. The packets were incubated at 27 ± 1°C and ≥ 80% RH for 24 h. Dead and live larvae were counted. Four replicates were performed for each concentration.

### Statistical analysis

The data were initially transformed to log(X), normalized and then nonlinear regression were calculated to get the inhibitory concentration (IC_50_) of *H*. *contortus* larval exsheathment and the lethal concentration (LC_50_) for *R*. *microplus* larvae for both extracts using GraphPad Prism 7.0 software (GraphPad Inc., San Diego, CA, USA) with respective 95% confidence intervals (95% CIs).

## Results and discussion

**Fig 1:**
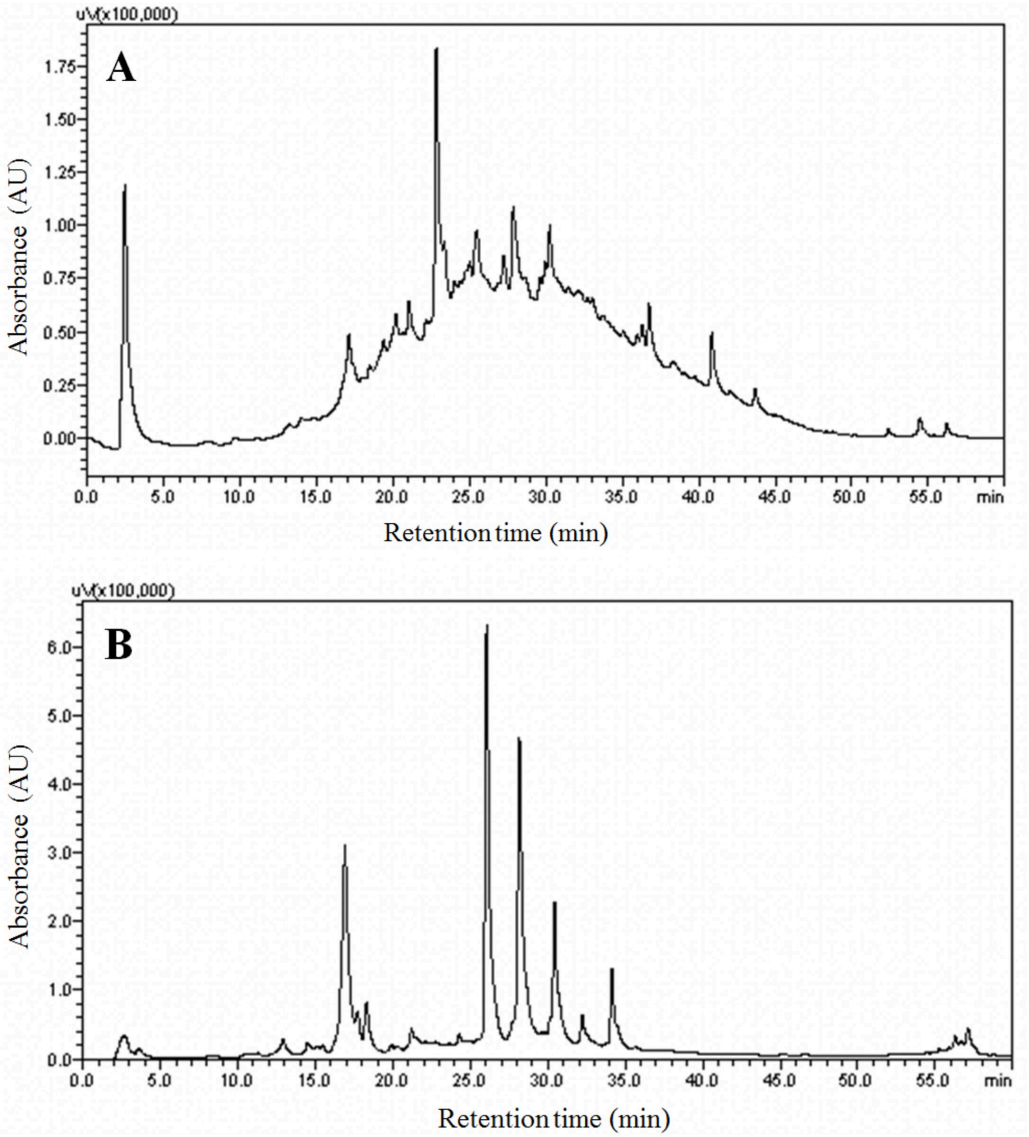
HPLC-UV chromatogram (254 nm) of *Ipomoea imperati* hydroethanolic extracts. a. chromatographic profiles of leaves. b. chromatographic profiles of stolons.

The present study evaluated the antiparasitic effects of separately prepared hydroethanolic extracts of *I*. *imperati* leaves and stolons against larvae of the nematode *H*. *contortus* and of the tick *R*. *microplus*. The leaf and stolon extracts of *I*. *imperati* showed different chromatographic profiles. The *I*. *imperati* leaf extract exhibited 12 secondary compounds, with various flavonoid aglycones and condensed tannins (Table 1), as evidenced by overlapping peaks and low resolution in reverse phase (Fig 1A). Eleven secondary compounds were found in the stolon extract, with a predominance of phenolic acids and glycosylated flavonoids and high resolution in reverse phase (Fig 1B). The compounds found in the *I*. *imperati* leaf extract confirm the results obtained by Jardim et al. [13], whose preliminary phytochemical screening also showed the presence of tannins and flavonoids. However, the secondary compounds present in the leaves and stolons of *I*. *imperati* were identified for the first time in the present study. Chemical characterization of plant extracts is fundamental for understanding pharmacological effects and for the validation or discovery of biologically active compounds [26, 27].

**Table 1:** Identification of compounds in *Ipomoea imperati* leaf and stolon hydroethanolic extracts by LC-ESI-IT/MS.

Part	[M-H]	MS^n^ fragments	Compound
Leaves	285	267; 241; 227	Kaempferol
	283	268	Acacetin
	447	429; 357	Isoorientin
	433	301; 271; 151	Quercetin-xyloside
	297	267; 161	3',7-Dimethoxy-3-hydroxyflavone
	463	301	Isoquercitrin
	431	313; 283	Isovitexin
	595	433; 301	Peltatoside
	594	355; 149	Pelargonin (antocianin)
	609	447; 301; 285	Rutin
	575	423; 289	Procyanidin A-type Dimer
	491	476; 329; 215	Iristectorin A
Stolons	173	155; 111	Shikimic acid
	191	173; 93	Quinic acid
	207	199; 162	3,4-Dimethoxycinnamic acid
	297	211; 161	3-hydroxy-3ʹ, 4-dimethoxyflavone
	353	191; 173	Chlorogenic acid
	431	287; 165	Kaempferol-rhamnopyranoside
	315	299	Isochlorogenic Acid A
	594	594; 450; 354	Pelargonin (antocianin)
	593	285; 227	Kaempferol-Glucoside-Rhamnoside
	415	254	Daidzin

**Fig 2:**
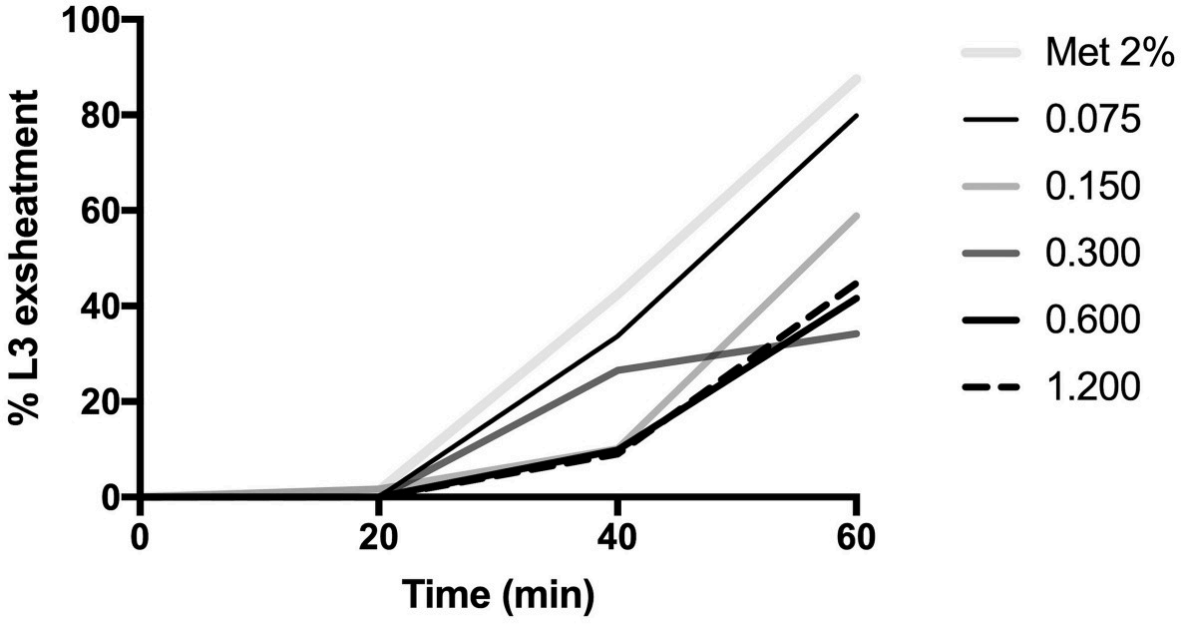
Effect of the *Ipomoea imperati* leaf extract on exsheathment of artificial third-stage larvae (L_3_) *in vitro* of *Haemonchus contortus*.

Anthelminthic activity was evaluated by a larval exsheathment inhibition assay to determine the potential of the extract to inhibit sheath loss in third-stage larvae (L_3_) of *H*. *contortus*. Preventing sheath loss in *H*. *contortus* larvae breaks the larval life cycle [24] and does not allow establishment of an infection in the host [28]. This test is a sensitive tool for detecting anthelminthic activity in plant extracts and enables comparisons with other species [29]. In the present study, the hydroethanolic extract of *I*. *imperati* leaves showed satisfactory results in larval exsheathment inhibition, with a linear reduction in exsheathed larvae with increasing dose (Fig 2) and an IC_50_ of 0.22 mg/mL (Table 2). Similar values are demonstrated by Alonso-Díaz et al. [29] for the tropical plants *Leucaena leucocephala* (Lam.) de Wit. and *Brosimum alicastrum* Sw. that presented inhibitory concentrations (IC_50_) of 0.21 mg/mL and 0.29 mg/mL, respectively. Other tropical plants evidence total inhibition of the larval exsheathment in 0.3 mg/mL [30] and 1.2 mg/mL [31].

**Table 2:** Effective concentration of *Ipomoea imperati* leaf and stolon extracts required to inhibit exsheathment of 50% of the larval population of *Haemonchus contortus* (IC_50_). CI_95%_ denotes confidence interval.

Species	Plant part	IC_50_ (mg/mL)	CI_95%_	R^2^
*Ipomoea imperati*	Leaf	0.22	0.18–0.26	0.84
	Stolon	> 1.2	*	*

* = Unable to determine because the IC_50_ values are below the minimal detection limit of the assay.

In contrast, all *H*. *contortus* larvae were able to exsheath, even at the highest concentration tested, in the *I*. *imperati* stolon extract, demonstrating the difference between these extracts (Table 2). Extracts of several plant species are rich in phenolic compounds that inhibit nematode exsheatment [32, 33]. However, few studies have reported different activities among parts of the same plant [34].

The results of screening for compound identification show primarily nonpolar compounds in the leaf extract and polar compounds in the stolon extract (Table 1). A higher nonpolar content can positively influence anthelmintic activity effectiveness [35]. This is due to the abundance of lipids in the cuticle of nematodes, providing greater transcuticular permeation capacity to less polar substances, resulting in stronger anthelminthic activity [36]. Thus, the nonpolar profile of the leaf extract might have contributed to enhance penetration and consequent activity in the larval exsheathment assay.

Another marked difference between the extracts is the presence of tannins (procyanidin A-type dimer) in the *I*. *imperati* leaf extract, but with no evidence of such molecules in the stolon extract (Table 1). Several studies have reported the anthelminthic effect of certain plants due to the presence of tannins among the chemical constituents of extracts, observing a reduction in or absence of biological activity in extracts due to the addition of compounds such as polyethylene glycol or polyvinyl polypyrrolidone that counteract tannins/phenolics [24, 31, 37, 38, 39, 40]. The action of tannins is mainly revealed by larval exsheathment inhibition [41], which may be responsible for the nematicidal activity observed for the *I*. *imperati* leaf extract. The influence of tannins in inhibiting sheath loss in L_3_ of *H*. *contortus* has been attributed to the strong similarity with amino acids (proline and hydroxyproline) present in the proteins that compose the structure [28]. Formation of such a complex would promote ultrastructural modifications in the sheath, preventing exchange between the environment and larval tissue and causing asphyxia or toxicity [42].

Structural differences such as condensed tannin polymer size and major tannin type (procyanidins and prodelphinidins) interfere with anthelmintic activity [43]. In addition to the procyanidins found in the leaf extract, which is less active on nematodes, we found a large amount of flavonoids with recognized anthelmintic potential, such as kaempferol, isoquercitrin (derived of quercetin), and rutin compounds (Table 1) [44, 45, 46]. A synergistic anthelminthic effect between tannin and flavonoids has been reported [33, 47] and maybe responsible for the anthelminthic activity of the *I*. *imperati* leaf extract in the present study.

Although other species of *Ipomoea* are used as antihelminth treatment [12, 48, 49], few studies have demonstrated bioactivity. Indeed, the *in vitro* effectiveness of *I*. *imperati* has only been shown in a study on *H*. *contortus* larvae, and only *I*. *staphylina* and *I*. *carnea* have been investigated for anthelminthic activity against earthworms [50, 51]. These data signify the importance of this genus in worm control, which should encourage the exploration of new species, preferably those that already have a history of use in popular medicine.

Regarding *R*. *microplus* tick assays, the *I*. *imperati* stolon extract presented low activity (35.7%) at 25 mg/mL. We suggest that this minor activity is due to compounds derived from cinnamic acid (quinic acid and 3,4-dimethoxycinnamic acid) (Table 1), which has been associated with acaricide effects [52].

## Conclusions

This study is the first to assess the anthelmintic and acaricidal activities of *I*. *imperati*. Data reported give no evidences to support the acaricidal property of *I*. *imperati* in controlling *R*. *microplus*. But, confirm promising potential of *I*. *imperati* leaves hydroethanolic extract against *H*. *contortus*. This effect could be due to its secondary compounds presents in this extract, such as procyanidin, kaempferol, isoquercitrin and rutin.
